# Professional values education for undergraduate nursing students: developing a framework based on the professional values growth theory

**DOI:** 10.1186/s12912-024-01743-0

**Published:** 2024-04-02

**Authors:** Jialin Li, Xiaohan Li

**Affiliations:** School of Nursing, China Medical University, Shenyang, China

**Keywords:** Education framework, Professional values, Nursing students, Theory derivation

## Abstract

**Background:**

Education has been recognised as necessary in forming and internalising professional values. The system and instructors' content in existing educational institutions focus on developing students' knowledge, skills and practices. Still, the development of values has yet to achieve significant effects, leading to a crisis in students' professional identity.

**Aims:**

To construct a professional values growth theory for undergraduate nursing students and develop a corresponding education framework.

**Methods:**

Through the review, some databases(PubMed、CINAHL、Web of Science、Wiley and Google Scholars)were searched using a systematic search strategy to collect relevant literature on professional values education. Based on the nursing professional values growth theory (Li and Li, Nursing Ethics In press, 2022), a theory of professional values growth of nursing undergraduates was developed using the method of theory derivation. Two rounds of expert meetings were conducted to review and revise an education framework of professional values of nursing undergraduates derived from that theory.

**Findings:**

A total of 10 studies were included. The contents of two themes were analysed: theories and models and the current status of the professional values development of nursing students. The resulting professional values growth theory for undergraduate nursing students consists of five parts: key aspects, decisive opportunities, drivers, embodiment (humanistic sentiments, moral emotions), and outcomes. A total of five experts in the relevant fields were invited to this study. After two rounds of expert meetings, an education framework for undergraduate nursing students was finally developed, which consists of four parts: education objectives, education process and content, environment and conditions, and evaluation.

**Conclusion:**

The education framework developed in this study has practical implications for the development of professional values of undergraduate nursing students, providing educational strategies and methods for the growth and internalisation of professional values of undergraduate nursing students.

**Supplementary Information:**

The online version contains supplementary material available at 10.1186/s12912-024-01743-0.

## Introduction

With the rapid advances in various fields such as technology, economy, and culture, healthcare from an international perspective has witnessed the inclusive coexistence of multiple cultures and a deepening trend of globalization. China's nursing workforce has grown in numbers and qualifications since 2016. In 2020, China's National Nursing Career Development Plan (2021–2025) emphasises the need to fully mobilise the nursing workforce and improve the quality of nursing services [1]. The cultivation of nursing professionals has always been an essential mission of higher education institutions, in which the cultivation of nursing professional values has become an important element in the training of undergraduate nursing students [2]. In the context of global multiculturalism, the formation of good nursing professional values can not only enhance the professional satisfaction of undergraduate nursing students [3] and improve ethical decision-making ability [4], but also shape the profession's social image. Based on previous theoretical research, this study develops a framework for cultivating professional values among undergraduate nursing students and provides educational strategies and methods for the growth and internalisation of professional values among undergraduate nursing students.

## Background

Values are the deeper meanings and perspectives on something that are developed by one's needs and experiences; they are ideologies of social life that are formed and maintained over time by individuals through their life experiences, and are not affected by changes in the organisation's social environmen [5]. Values influence individuals' behaviour in everyday life and guide their evaluation of themselves and others, choices, and actions [6]. Professional values are a specific choice of values based on the connotations and characteristics of a profession and are used as an element in shaping the identity of a professional group [7]. Although there is no unified definition of professional values to date, more scholars have stipulated and explained them [8, 9]. It is also clear that professional values are a product of the inevitable formation of the socialisation process [10].

In nursing, values become a breakthrough in establishing a unified and accepted model of behaviour within the profession [11]. In 2016, Bonnie J. Schmidt et al. conceptualised nursing professional values through Walker and Avant's concept analysis, which identified nursing professional values as Human dignity, Integrity, Altruism and Justice, which are crucial professional nursing principles and frameworks for clinical practice and assessment [12]. Nursing professional values are closely related to three themes, namely patients, the profession and society, and are agreed upon in a global perspective based on ethical guidelines [13]. Meanwhile, Derek Sellman identified rigid value propositions among administrators and policymakers at all levels of health care, contributing to the marginalisation of nursing discourse, as well as the subordination of nursing to the health care system, which has marginalised and homogenised its values [14]. A study by Cho S.H et al. also found that in the social–historical process, the deep-rooted stereotypes formed about the image of nurses have influenced students' perceptions and formation of professional values in choosing whether to pursue a nursing profession [15]. Therefore, when the socialisation process of nursing students is fraught with a professional identity crisis, transforming their perceptions of nursing from a widespread perception of the profession to an understanding of the professional profession can be challenging and obstructive.

In China, nursing students are demanding about the geographical location of their employment, the salary package, and the number of night shifts, and hospitals and local governments need to adopt more material incentives to be able to attract more nurses to work in rural areas [16]. Yi qi feng et al.'s study also found that nursing students generally have employment anxiety [17], and that professional identity is a key factor influencing the employment intention and career planning of nursing students [18]. This highlights the importance of nursing education in fostering good professional values and socialisation to prepare students for the demands of the nursing profession and bridge the gap between theory and practise. Students' beliefs that 'I am doing this to serve the health of others' and 'I deserve to be paid well for my work' often coexist, requiring education to guide, assist, and adequately support them in this process. Kaya et al. investigated the changes in undergraduate nursing students' professional and personal values over four years of study [19]. They found that the differences in the measures of professional values between the four years of undergraduate nursing education were statistically significant, showing that education has been identified as a crucial prerequisite for forming and internalising professional values.

Instructors often focus more on the external norms reflected in professional values and less on their complex internal nature [20], making teaching values easier. However, this strategy fails to provide nursing students with emotional education and prevents them from forming and internalising professional values. The existing institutional system and the content of lessons focus on developing students' knowledge, skills, and behaviours. Still, the development of professionalism and values has yielded insignificant results, thus creating more of a professional identity crisis [21]. Students can gain satisfaction from technical mastery, but working in nursing solely for money and status is not nursing. It may even lead to questioning the fairness of external benefits [14], creating a dilemma for nursing staff. Therefore, on top of the emphasis on technical competency in professional education, there is a greater need for professional values to be involved in professional socialisation in the form of internal rewards. Furthermore, personal experience influences individual values and behavioural choices. It argues that all values are not simply given from the outside but spontaneously formed in their growth and development and all socialisation experiences.

At present, research on nursing professional values has focused on the current situation and influencing factors of professional values among nurses and nursing students, as well as the study of their correlation with several factors such as work environment, job satisfaction, self-esteem, and nursing organisational climate [21, 22]. In-depth research has yet to be been conducted on the relevant development programmes and frameworks for nursing professional values of nursing students at the undergraduate level. Our research group has developed nursing professional values growth theory based on grounded theory research in previous studies [23]. Therefore, the purpose of this study is to explore the current problems in professional values education in nursing undergraduates, and a theory specific to the growth of professional values in undergraduate students based on pre-theory is developed to guide the construction of an educational framework and address the challenges that may arise in future healthcare professionals' development.

## Aims

This study aims to develop a framework for the cultivation of professional values in nursing based on theories of professional values growth for undergraduate nursing students.

## Methods

### Theory derivation

In 2011, Walker and Avants introduced theory derivation in the methodology of theory construction, which refers to a theory (theory 1) that provides some new insights from an area of interest (domain 1). The theorist moves some of its content and structural features to another area of interest (domain 2), forming a new theory (theory 2).Theory derivation could notice two different domains and dimensions of similar phenomena and enables the redefinition and transformation of theories from domain 1 to domain 2. Theory derivation is the process by which a set of related concepts or overall structures is transferred from one domain to another and modified to fit the new domain. Furthermore, Walkers and Avants also emphasised that at least some modifications in content and structure cannot be borrowed directly. Theory derivation is an iterative process, and the order of the steps may change during the research process, but it basically consists of five steps: (i) understanding the level of theoretical development in one's field of interest and being able to assess its scientific value; (ii) reading extensively in nursing and other fields while allowing imagination and creativity to run free; (iii) selecting the parent theory for a derivation; (iv) determining the content and structure of the parent theory; and (v) developing or redefining new concepts and statements from the content and structure of the parent theory [24]. Theory derivation requires the researcher to be familiar with the field of interest, and the new theory must be empirically tested to verify its relationship to actual reality [25].

The theory of professional values growth in nursing developed in the pre-study of our research group is applicable in a wide range of contexts, from nursing students to senior nurses. In order to construct a professional values education framework for undergraduate nursing students, this study cannot directly use the theory of professional values growth in nursing. Still, it should instead focus on the growth of professional values at the undergraduate education level in a context. Therefore, it is necessary to derive the theoretical framework on which this study is based, namely the professional values growth theory for undergraduate nursing students, based on the structure and content of the pre-study theory.

### Data sources

Given that a scoping review allows for a wide-ranging and comprehensive exploration of the research question, including the background, boundaries, and possible gaps [26], the inclusion of information collected from a systematic literature search to ensure the completeness and accuracy of the included literature, and the emphasis of the paragraph on an overall theme, it was chosen as the data collection method for theory derivation and the development of education frameworks.

A systematic search is conducted to understand the relevant theories and educational frameworks for professional values in nursing. The following two main questions were addressed: (i) What are the relevant theories and models of nursing professional values? (ii) What is the educational framework for professional values education for nursing students?

1. Search strategy

The search terms in this study were developed according to PCC [27], where population (P) is nursing student or undergraduate, concept (C) is nursing professional values, and context (C) is the theory, training programme, education framework. Two databases (CINAHL and PubMed) were first used to understand the basic research in the field and to identify further relevant search terms. The search terms used in this study were "professional", "profession", "nurses", "values", and "training". "values", "student", "undergraduate", "theory ", "education", and "training". The search databases included PubMed, CINAHL, Web of Sciences, and Wiley. Also, grey literature was accessed through the official nursing website, Google Scholars. The search time interval was 2000 ~ present. The search was conducted in March 2022. (See Appendix 1 for examples of the search strategy).

(2) Inclusion and exclusion criteria

Inclusion criteria: articles published in English with access to the full text; the population was nursing students, with no restrictions on an academic background; the literature included theoretical models or training programmes for nursing professional values. Exclusion criteria: nursing students in the study population were midwifery.

(3) Selection process

After excluding duplicates from the retrieved literature, one researcher (LJL) first screened the literature by reading the titles and abstracts. Then two researchers (LJL LXH) read the titles and abstracts of all the literature initially screened on this basis and jointly discussed the deletion of literature that was not relevant to the study. The two researchers then independently read the full text and excluded literature that did not meet the inclusion criteria of the study, and finally reached a consensus on consensus on the literature to be included in the study.

(4) Collation and analysis of literature

In this study, the information was collated according to the literature extraction form designed in advance, which included author, year of publication, country, type, aims, methods, population, instrument, data collection methods and results. The literature extraction was first carried out by one researcher for the first three included papers and adjusted the literature inclusion form, after which two researchers completed it independently and discussed it after completion. According to the research questions, the analysis method used was thematic analysis.

### Framework formation process

This study ‘s theoretical foundations included the professional values growth theory developed by the pre-study and constructivist learning theory. The education framework was developed through group discussions considering feasibility, including the following four parts: objectives, content and methods, environment and conditions, and evaluation.

Five experts in nursing ethics and education fields (see Appendix 2) were selected to discuss the cultivation framework in two rounds of expert meetings. The cultivation framework was revised and adjusted according to the experts' opinions and suggestions. The first round of expert meetings was conducted online. A consultation questionnaire was sent to the experts by email before the meeting, including a letter to the experts, a professional values theory and cultivation framework for undergraduate nursing students, and an expert information list. The second round of the expert meeting was used to review the revised framework by email.

### Ethical consideration

The Ethics Committee of China Medical University approved this study under approval number [2022] 86.

## Findings

### Literature review results

**Fig. 1:**
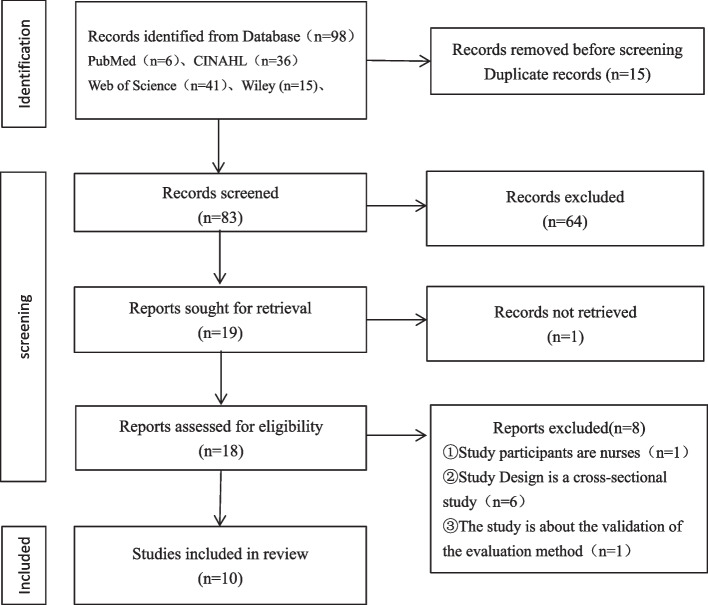
PRISMA-ScR flow diagram

A total of 98 pieces of literature were retrieved, 15 duplicates were removed, and the remaining 83 were screened, of which 64 had titles and abstracts that did not meet the inclusion criteria for this study. The remaining 18 were read, and 8 were removed. The final 10 articles were included in this study, see Fig. 1, of which 5 were reviews, 8 were experimental studies, and 1 was an action study, the details of which are in Appendix 3 and 4.

A total of six nursing professional values theories or models were included, collated, and summarised in this study. The context of their theory construction, the theories' paradigmatic origins, and the theories' internal dimensional analysis were analysed according to Meleis' theoretical analysis strategy, as shown in Table 1.

**Table 1:** Analysis of theories and models included in the study

Meleis' theory analysis		Five values of the nursing profession from the American Association of Colleges of Nursing (AACN)	Model of factors and corollary values influencing professional values development	The Professional Values Model in Nursing	Moral agency of nurses model	The process of attaining, enacting and socialising values	the Values Based Enquiry model
**Theorist analysis**		The American Association of Colleges of Nursing AACN works to establish quality standards for nursing education; assist schools in implementing these standards; influence the nursing profession to improve health care; and promote public support for professional nursing education, research and practice	Darlene Weis is the founder of the Nursing Professional Values Scale and Professor Emerita at Marquette University. Mary Jane Schank, an American nursing educator, is a member of the American Nurses Association, the National League for Nursing, the Association for Research in Nursing Education and the Midwest Nursing Research Association	The researcher is currently working as a research assistant at the Department of Nursing, Akdeniz University, Turkey, with research interests in principles of nursing, nursing, and critical care medicine	One of the researchers, Barbara J Daly, a nursing educator and nursing philosopher, led a task force from 1996 to 2001 that rewrote the American Nurses Association's code of professional ethics	The researcher team includes four people with health-related majors, including Sastrawan, a postgraduate student, and Jennifer, an associate professor at the University of Melbourne with a Ph.D. in education. Gabrielle Brand is a faculty member in the School of Nursing with expertise in qualitative research, and narrative medicine. Gulzar Malik is a senior lecturer in nursing at La Trobe University with a specialization in acute and critical care	The study involved the Centre for Curriculum Development at the University of Southampton,,UK and the researcher is a Principal Teaching Fellow in the School of Health at the University of Southampton. She has extensive experience in curriculum development and design and is currently the Programme Leader for the Pre-registration Nursing Programme for the Master in Nursing
**Origin of the paradigm**		Code for Nurses issued by the American Nurses Association	Code for Nurses issued by the American Nurses Association	(1) Watson's theory of caring (2) Pender's model of health promotion	(1) Self-esteem theory (2) Value theory The two theories above argue that (i) the self consists of multiple unique and meaningful identity standards and guides decision-making and behaviour. (2) Describes a potential internal conflict when a situation creates inconsistencies in identity standards or internalised value structures	A paradigm for constructivist grounded theory	Theoretical foundations: virtue ethics and values-based practice (1) Virtue ethics is based on Aristotle's theory that virtues can be developed from habits and that character can be conceptualised as those contribute to the achievement of specific goals (2) Values-based practice is a healthy way of practising because self-awareness and the skills needed to recognise and respond to the values of others are required in clinical practice and decision-making
**Internal Dimensions**	**rationale on which the theory is built**	Based on the 2001 American Nurses Association Code of Ethics and is described around the five main concepts of altruism, autonomy, human dignity, integrity, and justice	Interpretive propositions (1) The theory was developed before the American Association of Colleges of Nursing identified the fundamental values in baccalaureate nursing education in 1998 (2) Scholars at the time believed that nurses needed to learn cognitive and psychological domains and emotional skills and that these three areas were essential to professional development and professional socialisation	Existence propositions Theoretical assumptions: (1) Individuals attempt to create healthy behaviour that demonstrates personal values and will be influenced by personal experiences (2) Individuals always show change and development and have the possibility to express themselves (3) Individuals influence and are influenced by their environment at all stages of their lives (4) The higher the professional values of nurses, the higher their job satisfaction and quality of care	Interpretive propositions Theoretical assumptions: (1) Nursing professional values are based on the 2008 ANA Code, learned and internalised through environmental and social exposure (2) The three main premises of the internalisation of nursing professional values are that knowledge of values is personal (cognitive), the importance of values is subjective and relative to other held values (affective), and values can be maintained when perceived to be in flux (behavioural) (3) The identification of ethical dilemmas initiates a process of self-validation that requires a reflection of value identity, fulfilling of role expectations, and the skills and resources available for conflict resolution (4) Confidence in ethical decision-making is a subjective assessment of one's ability to identify conflicting values of interest, identify role expectations, reflect on skills and knowledge readiness, and assess one's ability to 'do the right thing' in a situation (5) Self-confidence in ethical decision-making plays an essential role in an individual's level of self-esteem (6) Self-esteem results from integrating personal and professional values, a motivation to maintain a commitment to identity standards, and a buffer against moral distress when values are inconsistent	Interpretive propositions The development of fundamental values for nurses contributes to developing of their value system. Interviews with nursing managers and other stakeholders were used to understand the various influences on their values, to describe events and past experiences and reflections that have influenced their values, and to ask questions such as "How did you develop as a professional nurse?", "How have you developed your personal, professional identity/values/views/attitudes?"	Existence propositions (1) The UK Nursing and Midwifery Council set new standards for nursing education in 2010, suggesting that graduates of nursing and midwifery should be of good character on entry to registration (2) Educators cannot directly endow students with good character but can motivate them to follow their paths in life
	**system of relations**	/	A field approach The model describes the role and influence of education, professional processes, and institutions/services in developing professional values, as well as the core value attributes of professional values. The relationships between the variables are described, the role of education as a driver of professional values development is affirmed, and it is explicitly argued that nurses' practice behaviours in clinical practice and their interactions with the organisation all impact on the formation of professional values	A monadic approach The theory focuses on explaining professional values through three basic concepts	A field approach The theory describes the relationship between nursing professional values, self-esteem, and ethical decision-making, thus reflecting how ethical decision-making and self-esteem can be influenced and changed at different stages of professional value enhancement	A monadic approach The process theory is based on how nurses form their professional values. The three stages of the social process of professional values formation are described, and the relationship between each stage is explained	A monadic approach The core of the model emphasises professional values of self-awareness, caring and compassion, and awareness of the values of others. The model specifies how professional values in nursing education should be internalised in future students who become nurses and midwives, focusing only on the characteristics of students' internalised professional values and is a consideration of a single factor
	**Content of the theory**	/	Micro theory	Micro theory	Micro theory	Micro theory	Micro theory
	**Theory beginnings**	/	Constructive beginning The theory is presupposed to be hypothetical, i.e. thinking about the relationships between variables in terms of assumptions. Ten premises of values formation are stated in the theory, including that values occur as practice progresses and may be subject to stagnation and imbalance; that there are key stages that facilitate and hinder the development of professional values; that preparation and purposeful experience are key factors in the development of values; that the personal values of the student component are aligned with nursing values; that the nurse-patient relationship and its values should be encapsulated in professional nursing education. Values related to social issues should be ranked as a secondary position, e.g. activism; higher education promotes values development; mentoring by individuals who have internalised values helps develop the professional values commitment of others; policy development, the nature, and scope of nursing practice should also be a nursing professional concern; professional values are reflected in action, etc	Principle theory beginning The nursing profession's values developed in this study contain three foundational concepts (1) Personal values: (i) Personal characteristics, experiences, perceived health and illness status, needs, priorities, environment, and society play an important role in the acquiring of values and the creation of value structures. (ii) Personal values guide the choices and priorities of individuals in the decision-making process. (iii) Values have a significant impact on individuals' decisions about their needs when they are healthy or ill. (iv) Values play an important influence in the individual's involvement or rejection of their care (2) The values of the nursing profession were identified by the American Association of Colleges of Nursing and, based on a review of the literature, have been developed as truth, integrity, altruism, autonomy, equality, human dignity, and aesthetics (3) The goal of the nursing profession is to provide effective and high-quality care based on patient's individual needs, using constantly updated knowledge	Constructive beginning It has been demonstrated that there is a relationship between self-esteem, ethical decision-making, and internalisation of professional values, so the researcher has confirmed that the relationship between the variables, in theory, does exist by hypothesizing and testing the relevant variables	Principle theory beginning The theory is based on the collection and analysis of data through the research methodology of constructivist rooted theory, which utilises an inductive approach to theory generation and proposes the need to go through three stages of realising values, formulating values, and socialising values	Constructive beginning The core propositions of the theory consider self-awareness, care, and compassion and the identification and understanding of the values of others to be central to students' development of their future identity as nurses and midwives (1) Students must be aware of their values and behaviours (2) Be mindful of the professional values of care and compassion (3) Reflect on how to relate to and respond to the values of others
	**Scope of theory**	/	Situation-specific theory	Situation-specific theory	Situation-specific theory	Situation-specific theory	Situation-specific theory
	**Goal of a theory**	Describe the five professional values based on the code of ethics for nurses	(1) Reflect on how education, professional practice, and the profession influence professional values (1) Reflects the social nature of the profession and its responsibilities to the public (3) Guide nursing educators in the preparation of future nurses (4) To guide providers in developing standards and assessment measures to advance the profession	(1) Describe personal characteristics, values, and experiences to provide personal care based on the whole person (2) Describe nurses' personal and professional values and clarify themselves (3) Improve nurses' professional values and job satisfaction (4) Provide quality care and increase patient satisfaction with care	Describing and validating the relationship between nursing professional values, self-esteem, and ethical decision-making will help educators to clarify the relationship between self-esteem development and the internalization of professional values	(1) Reflects on the basic processes nurses go through in forming their value systems (2) Reflects on how professional nursing values are formed and socialized into nursing practice	(1) To identify how to develop nursing and midwifery graduates with good character, provide students with intrinsic motivation, help them develop critical thinking, and develop their self-efficacy and moral agency (2) Provides support for a theoretical framework for ethics education developed under the Nursing Education Standards of the Midwifery and Nursing Council of the United Kingdom (3) Reflects the explicit conceptual nature of education with a broader nursing profession
	**Context of a theory**	Knowledge of disorder The fundamental values of the nursing profession are set out in the Code for Nurses issued by the American Nurses Association, which emphasises that a code of professional ethics is essential to practice	Knowledge of order The model assumes that education, service institutions, and professional practice behaviour influence professional values. (1) Professional values are an important part of professionalism and reflect the nature of society and responsibility to the public. (2) Education, service, and the profession play an important role in professional development. That is, learning in the formal education species must be engaged by service professionals to be more fully integrated into the profession. (3) A common, and shared core value system is a hallmark of excellence within the organisation and the organisation plays an important role in enhancing the value of personal growth	Knowledge of order The theory suggests that professional values include three underlying concepts: personal values, professional nursing values, and quality of care	Knowledge of control In this model, high or low professional values in nursing affect professional identity standards. When an individual in an ethical dilemma has their ethical decision-making influenced by positive and negative circumstances, this allows for moral self-confidence changes, affecting self-esteem. In contrast, self-esteem can impact professional values and ethical decision-making	Knowledge of control The three stages developed in this study, of which the first stage is the realisation of values, is a theory that emphasises the important role that the family plays in shaping nurses' personal values, particularly in the early years of the nursing profession. The second stage is the formulation of values, which theorises that nurses' values are reactivated and reinforced during their professional training. The third stage is the socialisation of values, which suggests that nurses often share and continue to develop their values informally with colleagues around them, encouraging nurses to revisit values that have been neglected	Knowledge of control (1) The theory consists of a core section and prompt questions, which are a collection of three questions for instructors to promote the professional values of nursing students (2) The prompt questions promote the proposition in the theory that the formation of good professional values in nursing students requires the individual student to want to become a nurse or midwife in the future rather than 'learn' to do what it takes to become a nurse or midwife (3) Prompting questions to include three categories, namely critical and analytical skills, internal motivation, self-beliefs, and self-efficacy, based on which students can engage in reflective practice, and the researcher also describes and gives examples of how the three categories of prompting questions are used in the text
	**Abstractness**	Low abstractness	Low abstractness	Low abstractness	Low abstractness	Low abstractness	Low abstractness
	**Method of theory development**	/	It was developed based on literature research	Based on literature research, clinical experience, and the development of the researcher's knowledge and perceptions	A synthesis of self-esteem and values theories was used to form the theory	It was developed from an approach based on Charmaz's constructivist grounded theory. Data was collected through in-depth personal interviews, focus group interviews, anecdotes, and reflective writing and analysed through a continuous comparative approach to form the theory	Metaphors. Drawing on The Wizard of Oz, Dorothy encounters the Tin Man, the Cowardly Lion, and the Scarecrow, all of which are Dorothy's weaknesses, on her way home along the Yellow Brick Road. Eventually, without the help of the Wizard, Dorothy returns home and discovers that she has always possessed these personal qualities that she thought she did not have in the past. Therefore, the researcher describes the character development process as the 'yellow brick road'

The content of the existing nursing professional values education framework is summarised according to the included literature. The analysis revealed that existing nursing professional values education is divided into two forms of organisation: classroom teaching, where the school sets the professional values course content and syllabus, and extra-curricular teaching, where students choose whether or not to participate in extra-curricular teaching programmes, such as intercultural education and international service projects. See Table 2 for details.

**Table 2:** Nursing students' professional values development studies summary

**Items**	**Contents**
**Period of education**	1 week, 4 weeks, 8 weeks, 4 years
**Target participants**	Undergraduate nursing students, post-secondary nursing students
**Form of organisation**	Values development courses, hidden curriculum, film teaching activities, job experience activities, thematic practical activities, transcultural nursing education, international service learning projects
**Education methods**	Lectures, seminars, group discussions, oral presentations, reflective diaries
**Education strategy**	(1) Components of professional values education: (i) early introduction of values in nursing education; (ii) motivating students; (iii) nature of values; (iv) cultural values; (v) examination of personal values; (vi) clarification of the five core values (2) Professional values model: (i) personal values; (ii) professional nursing values; (iii) quality of care (3) Core values education: (i) "human dignity" in the first semester; (ii)"integrity" in the second semester; (iii) "autonomy" in the third semester; (iv) "altruism" in the fourth semester; (v)"Altruism" in the fourth semester; (vi) "Social justice" in the fifth semester (4) The seven pillars of "giving voice to values": values, choices, norms, purpose, self-knowledge and alignment, voice, reason, and rationalisation (5) A model for teaching values-based inquiry learning: self-awareness, caring and compassionate professional values, and awareness of the values of others
**Education content**	(1) Group discussion: conceptual definition of professional values, sharing stories of altruistic behaviour, discussion of social justice issues (2) Experiential activities: sensory substitution experience, participation in a mock job application exercise, acting as a practical room manager, clinical simulation, watching a film related to professional values in nursing, and creating a living advance directive (3) Teaching activities: participation in study tours abroad, conference preparation, ethics courses, cross-cultural nursing courses, analysis of disciplinary incidents among nurses, clinical apprenticeships, case studies, sharing of public figures' deeds, and public health nursing courses (4) Social activities: participation in volunteer activities, etc
**Evaluation methods**	(1) Qualitative evaluation methods: (1) Focus group interviews; (2) Values clarification exercise questionnaire including the following questions: I believe the purpose of nursing is ……; I believe this purpose can be achieved through ……; I believe factors that may inhibit or make this purpose to be achieved factors include ……; I want to become a nurse because ……; I feel it is worthwhile when ……; I do not feel it is worthwhile when… …when (2) Quantitative evaluation method: questionnaire method (Revised Nursing Professional Values Scale, the Korean version of the Nursing Professional Values Scale)

### Theory derivative results

This study used the theory of professional values growth in nursing as a derivative of the parent theory through the Walk and Avant theory derivation. It developed a theory of professional values growth for undergraduate nursing students about the literature review findings.

### Theory description

**Fig. 2:**
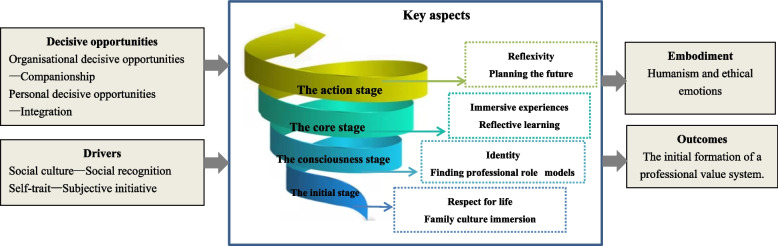
The professional values growth theory for undergraduate nursing students

The content of the professional values growth theory for undergraduate nursing students includes five parts, key aspects, decisive opportunities, drivers, embodiment and outcomes, see Fig. 2.

1. Key aspects
  The core of the professional values growth theory is the key aspect, which includes four stages: the initial stage, the consciousness stage, the core stage, and the action stage. However, undergraduate nursing students are in the professional learning stage and have not yet engaged in clinical nursing practice as nurses; therefore, their professional values at growth stage represent a preliminary professional value system.
  1. The initial stage: The initial stage of the growth of professional values is to build the foundation of professional values, including respect for life and family culture immersion. ①Respect for life: At the early stage of entering the nursing profession, students need to fully understand and appreciate the nature of the nursing profession, the pursuit of which is closely related to life. Respect for life is, therefore, a prerequisite for the growth of professional values in nursing, in which a sense of professional mission is developed, and a correct attitude towards human life and dignity is formed. ②Family culture immersion: virtue education in the family culture is part of forming their values. The family environment enables students to learn how to communicate and interact appropriately with others not part of the family. When the student is exposed to the nursing profession, the personal values acquired from the family culture that are compatible with professional values are perpetuated and integrated into the personal value system.
  2. The consciousness stage includes identity and finding professional role models. ①When students first encounter the nursing profession, they are full of unknowns about everything in the profession. However, individuals need to be understood, supported, respected, and helped within a whole group that has always been characterised by discourse: standards of practice, human attitudes, values, and goals; and to empathise with common interests, thus creating a sense of belonging within the group and among individuals. ②There will always be trusted predecessors or peers along the way, but the presence of role models is not the key to the growth of their professional value; instead, students should actively seek out role models and believe they can strengthen their personal future professional development. Role models convey professional values and career beliefs by word and example and translate their professional values into practice, thus exposing students to subtle influences.
  3. The core stage includes immersive experiences and reflective learning. ①Practical and applied professions require professional practice experience to gain a deep professional emotional education. Essential to the growth of professional values is the experience of authentic clinical nursing practice. Without an immersive practice experience, the humanistic and ethical sentiments in professional values will not be accurately felt. ②For professional values to grow and be internalised, constant reflection and self-reflection are required during the experience so that personal values that contradict professional values can be adjusted and attitudes and behaviours consistent with professional values can be gradually clarified. Active reflection and introspection are, therefore, inevitable processes in internalising professional values.
  4. The action stage is the final stage of the key aspects, including reflexivity and planning the future. ①As students grow in their professional values, their personal values are also influenced, thus bringing an "invisible sense of responsibility" to all aspects of professional practice. Secondly, when the professional values of nursing, which have grown through education, are "merged" with personal actions, the values assigned by the student's profession enable him or her to determine exactly when and how to present their professional roles and actions, even when temporarily removed from the professional environment. ②When students consider their profession or future career desirable, they are hopeful for the future and want to define their career aspirations further. Therefore, students create a professional future by setting short-term and long-term pursuits to give them professional confidence and motivation to move forward, taking purposeful action, and providing the possibility of other internalising professional values.
2. Decisive opportunities: The growth of professional values in undergraduate nursing students needs appropriate support and determinants. When individuals capture opportunities for the growth of professional values in nursing, they play a supportive role in their personal development and professional values. Furthermore, decisive opportunities are somewhat out of control but can be created through the environment and through one's actions and reflections to determine opportunities for professional values growth. These include organisational and personal opportunities.

Organisational decisive opportunities are the interpersonal processes of student-staff and student–student interaction within the campus culture and interpersonal environment in which students learn and live. These provide decisive opportunities to promote the growth of professional values in nursing. Companionship is the form of support that needs to be given in the interpersonal environment in which the student lives. Not only are students in the early stages of the development of their professional values, but they are also in the early stages of forming their value systems. They may encounter difficulties in their lives and studies that they cannot overcome.

Personal decisive opportunities are actions initiated and engaged in by individuals in the process of growing professional values. Such actions provide individual motivation for the growth of professional values and strengthen the link between personal feelings and professional values. As students become aware of professional nursing values, their growth requires them to use critical thinking, balance personal ideals with professional realities, integrate personal and professional emotions, gain self-recognition, and integrate self-fulfilment with professional development pursuits.

(3) Drivers: A variety of factors, particularly social culture and self-trait, influence and drive the development of students' professional values.

The recognition of the profession in social culture influences students' confidence in the value of the profession and their future careers in the related profession. The growth of professional values in undergraduate nursing students requires encouragement from social health policies, support for nursing talent development, access to professional autonomy, decision-making, and voice, as well as recognition and respect from patients and society.

In addition, the individual's subjective initiative in self-trait plays an important role. Individuals with solid subjective initiative who are able to proactively deal with and cope with the complex world and break out of the confusing situations they find themselves in can play a self-motivating role in the growth of their professional values. When students enter the nursing profession with a frustrating personal dilemma from stereotypes can cause them to become confused when it is vital to break through the harmful confusion and rethink the pursuit of nursing professional values through spontaneous introspection and reflection.

(4) Embodiment: Embodiment includes humanism and ethical emotions. Humanism refers to nursing professional values centred on human health, respect for human dignity and values, humanitarianism, consciously caring for others, loving others, and safeguarding the health of others. In the professional values growth of undergraduate nursing students, humanism is concretely reflected in fraternity, empathy, and care. Fraternity means that the nursing profession treats all patients and others with any health need with a spirit of non-discriminatory fraternity, treating them equally and giving care services to others selflessly. Empathy means putting oneself in the shoes of others, understanding and responding empathetically to the emotional problems caused by illness, and having the ability to empathise and feel empathy. Caring is motivated by "goodness" and demonstrates altruism in the care of patients. Caring is transferable and contagious, and when students receive care as part of developing their professional values, they will spontaneously want to give care to others in their profession or their lives.

Ethical emotions refer to the embodiment of moral obligations and ethical responsibilities in nursing professional values, which can transform different professional events into inner moral experiences and help individuals and groups to judge the rightness or wrongness of professional actions, specifically responsibility, discretion, kindness, sincerity, and equality. Responsibility refers to the ability of individuals in the nursing profession to take an active and positive attitude towards their obligations to maintain health and save lives and to fulfil them consciously. Discretion is the expression of ethical values in the form of self-discipline in nursing practice. Kindness means treating the lives of others well, providing timely care to help maintain or recover from health problems, and not harming others. Sincerity refers to an altruistic spirit of seeking truth and truthfulness in the treatment of those we serve. Equality refers to maintaining equality in health care by providing professional care to patients without discrimination or prejudice.

(5) Outcome: The growth and internalisation of professional nursing values continue with the change of role; therefore, at the stage of undergraduate nursing students, in conjunction with the requirements and objectives of talent training, the embryonic professional value system at this stage needs to be formed. In the process of receiving higher education, students are able to understand the nature of professional values, remain loyal to their profession, direct their clinical nursing practice activities in a spontaneous, active, and non-coercive manner, have their beliefs and value pursuits about the nursing profession ingrained in their hearts, take technical excellence and emotional firmness as the criteria for professional interpretation, and are able to infect others through their behaviours and attitudes in line with their professional values; this process is the key to the initial formation of a professional value system.

#### Education framework

Education framework includes four components, namely education objectives, education process and content, environment and conditions, and evaluation, as shown in the Table 3.

**Table 3:** Education framework of professional values for undergraduate nursing students

**Framework**			**Content**
Education objectives	Overall objective		Students can gradually grow and internalise their professional values and fully recognise and understand the humanistic sentiments and moral emotions embodied in professional nursing values Students can base their professional values on guiding their attitudes and behaviours during clinical practice to make them conform to professional ethics and morals
	Level objectives	Acceptance	• Students are able to recognise the meaning and significance of personal values and are aware of their importance and their relationship to personal growth and development; • With the instructor's guidance, students can attempt to describe and clarify their personal values; • Students are able to learn about the history of the nursing profession, express their concern for the profession, recognise outstanding figures in the nursing profession in China and abroad, and are willing to learn about their contribution and value to the profession through the instructor's explanation
		Reactivity	• Students are able to read books related to life values carefully, actively participate in reading and communication activities, and share their experiences; • Students are able to actively seek out their professional role models and try to imitate their behaviour; • Students recognise the future direction of the nursing profession and actively make study plans and career plans
		Forming values	• Students are able to recognise the nature of life, respect its meaning, and pursue its values; • Students are able to understand the values of the nursing profession and explain the connotation, meaning, and importance of nursing professional values; • Students are able to clearly articulate what they consider to be the order of importance of nursing professional values and the reasons for them; • Students are able to recognise the importance and significance of nursing professional values for professional development and form a stable • Students are able to clearly articulate the order of importance of their professional values and the reasons for them
		Organizing the value system	• Students are able to point out the relationship between professional nursing values and clinical nursing practice and are able to identify the embodiment of professional nursing values in practice; • Students are able to integrate professional values into their personal value system based on their attributes and solve dilemmas that they may face in clinical nursing practice based on their professional values
		Personalization of the value system	• Students are able to give judgments based on their value system about various situations in the nursing practice environment that is in line with their professional values; • Students are able to consciously provide nursing practice activities that are in line with their professional values
Education process and content	The initial stage—building the roots of professional nursing values		• In-class: life values education The nursing profession is concerned with the cycle of human life care, and it is therefore vital that students are guided to understand the nature of life, respect the meaning of life, and pursue the value of life in the early stages of the formation of professional values. Life values education needs to be combined with different nursing professional courses, such as Nursing Ethics, to realise the education of life values, life emotions, life responsibilities, and life meaning, thus laying the foundation for developing professional values
			• Out-of-class experiences: personal values awareness and clarification The instructor uses extracurricular activities to guide students in understanding the concept, meaning, and importance of values; identifying the relationship between values and personal growth and development; recognising the need to form and internalise professional values in nursing; and clarifying personal values. Specific formats include community service and personal growth story-sharing workshops
	The consciousness stage—enhancing the sense of belonging to the profession		• In-class: learning the history of the development of the nursing profession After entering the nursing profession, undergraduate nursing students begin to gradually get to know and understand the nursing profession and gradually form a professional identity through studying professional-related courses. In the consciousness stage of the growth of professional values, individuals need to form a sense of professional community and have a sense of belonging to the profession. Therefore, learning the history of the development of the nursing profession could help understand the development process and experience of the nursing profession, empathise with the contribution of nursing predecessors to the development of the profession, form firm professional beliefs, and lay the foundation for internalising professional values
			• Out-of-class: finding and sharing professional role models Students read autobiographies and books on nursing, find their role models, try to imitate and create role model behaviours, and share them with their classmates, who will then comment on and share them. Specific forms of sharing include an online micro-video applet as a sharing vehicle to showcase current role models in life that students admire and learn from and explain why,such as online short video personal role model sharing competition
	The core stage -Professional Experience Immersion and Professional Values Embodiment		• In-class: learning professional nursing values This stage focuses on learning the connotations of professional nursing values. Each values is taught to the professional curriculum, and each of the nine attributes is discussed and studied to how human dignity, care, altruism, responsibility, equality, justice, honesty, action, and autonomy are reflected in the work of clinical nursing practice. Teaching methods include special lectures, deed reports, service learning, film viewing, and group discussion
			• Out-of-class: case reflections on nursing professional values Scenario-based teaching is added to design clinical situations, problem situations, and discussion situations that address the relationship between nursing professional values and clinical nursing practice, demonstrating the nursing professional values in that situation, such as in the care of elderly or AIDS patients. Also, for students experiencing clinical internships, clinical experience sessions are held to share positive and challenging experiences of professional values demonstration that exists during practice
	The Action stage—integration of personal development and professional futures		• In-class: learning about the future development of the profession and career planning This stage is an active process in which the professional values of nursing are continually deepened and internalised. The future development of the nursing profession is explained, including specific topics such as nursing-related policies, future career development, and career planning design
			• Out-of-class: drawing a blueprint for personal future development The students will be able to draw up a blueprint for their future development by writing a personal plan in which they will set their personal career planning goals for the next 5 to 10 years, the conditions for completion, the process and ways to achieve them, and possible challenges and ways to overcome them
Environment and conditions	Internal conditions—reflection and breakthrough		In the growth of professional values in nursing, individuals must reflect continuously, break through the confusion of the growth phase of life, and integrate professional ideals with professional reality. This process requires an internal drive toward self-acceptance to develop professional values; therefore, the internal conditions are self-reflection by students and self-monitoring. With a critical understanding of the prejudices and stereotypes for nursing, they examine the profession's strengths, improve their professional practice skills, form professional self-confidence, and identify with nursing professional values, thus providing the internal conditions for the growth and internalisation of nursing professional values
	External conditions—a supportiveenvironment	Instructors	Instructors' companionship and positive motivation are essential in forming and internalising students' professional values. Class tutors are set up as needed to understand the individual situation of each student in the class and provide positive motivation. Care can be passed on to each other, and instructors need to be selfless in their care for their students, which permeates the student-instructor relationship in small ways. Instructors need to learn to think differently and be able to put themselves in the shoes of students at a stage of conflicting professional identities and try to help resolve them. At the same time, the instructor's image is also the image of the nursing profession in the students' minds, and its subtle influence is felt in forming their professional values
		The humanistic environment of the school	The school should strengthen the cultural construction of the college, set up decorations with the characteristics of the nursing profession, and highlight the cultural connotations of the nursing profession, such as placing a statue of Nightingale, hanging the college motto, and decorating the humanistic nursing gallery. Creating a supportive interpersonal environment where students and instructors help each other is also necessary
		The humanistic environment of the college	The college should create a humanistic environment that conveys respect and recognition for all professions and treats students of all professions equally
Evaluation	self-evaluation	Process evaluation	• One-to-one interview: Set up a stage interview outline for the four stages of the key aspects of the growth of professional values of undergraduate nursing students. ① The initial stage: What do you think it means to you to clarify your personal values? How do you think your personal values have influenced your understanding of the nursing profession? ②The awareness stage: How do you view the nursing profession now? What role do you think life role models play in the growth of your professional values? ③The core stage: What do you consider the professional values of nursing? How do you think nursing professional values are reflected in clinical practice? Please give a specific example. ④ The action stage: What do you think are the strengths of your future career in nursing? Please talk about how career planning has contributed to the growth of your professional values • Nursing professional values reflection diary: Based on the content of the attributes of nursing professional values, namely human dignity, care, altruism, responsibility, equality, justice, honesty, action, and autonomy, and taking into account the training process, the reflection diary topics are designed, including seven topics, namely "The value and dignity of life", "The meaning of care in the nursing profession", "How to face disciplinary incidents in nursing", "What should I do if I meet a patient with AIDS", "What is the meaning of caring in the health care field", "Talking about what is justice in health care", "What I know about the nursing profession", "My future career". The reflective diary provides a complete understanding of students' knowledge and understanding of professional values. Through students' written expressions, they can further internalise professional values in the process of self-professional values clarification
		Summative evaluation	• Nursing professional values measurement: This study used the Revised Nursing Professional Values Scale to measure professional values • Focus group thematic interview: The focus group interview included two aspects: first, what insights were gained about the learning of professional values, what elements were considered to be beneficial, what elements needed to be adjusted, and what other aspects were considered necessary to strengthen the understanding of professional values; second, talk about the understanding of nursing professional values and what personal, professional values are like
	Instructor evaluation	Process evaluation	• Observation method: To ensure the overall quality of professional values development, instructors need to evaluate the effectiveness of various teaching processes in infiltrating professional values into class and extra-curricular activities and complete a instructor observation manual. The manual is self-designed, and its content needs to be adjusted according to the range of professional nursing courses and the lecturer's requirements, mainly the educational objectives, educational process, teaching methods and strategies of the in-class, and the degree of student participation
		Summative evaluation	• Interview method: Instructors were asked to make personal summative evaluations of students throughout the professional values growth phase, explaining how instructors played a role in supporting students' professional values growth

## Discussion

The education framework is grounded in the theory of professional values growth for undergraduate nursing students and constructivist learning theory. The four stages, which comprise the key aspect of the theory, articulate the core development events and structure them in professional values education, fully demonstrating how the structured education framework is a process to achieve the growth and internalisation of professional values. Recent studies also argue that the current nursing education should clarify at which stage of development learning styles and professional values are developed [22]. The education framework under development integrates professional education and professional values cultivation throughout the undergraduate nursing program in an organic and seamless manner.. The four components of the education framework are permeably integrated with the existing education model for undergraduate nursing students. The content takes complete account of student-centred teaching and learning activities, uses available resources to mobilise students' independent participation, it also embeds cumulative, reflective, and collaborative learning among peers in the education framework to develop professional values. Therefore, based on the characteristics of the professional values growth theory for undergraduate nursing students, this study integrates professional values cultivation with undergraduate nursing education in an embedded way and adopts multiple perspectives to evaluate the cultivation effects comprehensively.

The nursing professional values growth theory for undergraduate nursing students is the basis for the formation of this education framework, which centres on key aspects and consists of four phases. In the initial stage, professional values growth is to develop attitudes and beliefs that honour life and are influenced by the subtle influence of family virtue education. Ingrid Snellman et al. [28] suggest that nursing's values are grounded in the principle of human worth and the power to experience a meaningful life, which emphasises the equality of human worth and respect for human rights, which is a reflection of the nursing professional values that identify with the equal right to life. In the consciousness stage, the formation of a sense of professional community is critical to the growth of professional values in nursing. For the power of role models, Bandura's [29] social learning theory points out that individuals need to learn through observation and imitate the exemplary behaviours and activities of role models to complete the process of individual social learning. The growth of professional values itself is the process of professional socialisation, which cannot be separated from social learning, and following role models and imitating them becomes the way to grow their professional values [30]. In addition, a study by Aimei Mao et al. [31] pointed out that nurses' professional identity is influenced by personal and professional values. In the core stage, in the process of growing professional values, people will actively or passively find out that there are contradictions in their understanding of the profession and find out how they can act by their professional values amid the contradictions, gradually clarifying their understanding of personal and professional values and clarifying what attitudes and behaviours are right and wrong in their professional values, what can be imitated, and what can be resolutely resisted, to achieve the internalisation of their professional values during their clarification process [32]. In the last action stage, the individual's professional values have the basic formation conditions; combined with other research on professional values [33], it can be found that the individual's interpretation of professional values ultimately needs to be embodied in the professional action. The formation of professional values needs to come from personal reflection and the support of the external environment. In the education process, students need to be aware of their personal and professional values and focus on their expressions of relevant behaviours, such as empathy and fraternity. Students engage in active or instructor-led reflection during ongoing values infusion education, identify the gap between themselves and practitioners in terms of professional values, and gradually grow and narrow the gap through continuous learning to develop mature professional values. A study suggests that the process of growing one's professional values is a journey of self-discovery and argues that what is "learned" does not influence what students "become" and "be" in the future [34]. At the same time, education also needs to provide space and opportunities for reflection, providing a supportive environment for instructors and peers to exchange and discuss. There are numerous strategic approaches to providing reflective teaching and learning, and a research demonstrates that film teaching can better help learners to enter professional contexts and also provide interventions and methods for their reflection [35].

In the current healthcare environment, the nursing profession often exists in a marginal or subordinate role, and active engagement in healthcare and demonstrating professional values are often weak [36]. It is more important for students to strengthen the development of professional values during education to deal with the threat of neglected professional values and the dilemma of ethical practice [37]. The development of professional values in the current research has usually been based on an unstructured hidden curriculum as a teaching model, but the hidden curriculum is defined as an informal course with no clear objectives and faces the problem that it is difficult to evaluate and give feedback on the effectiveness of the teaching in its unstructured framework. Therefore, a formal emotional education framework should be established to develop professional values and integrate them into the undergraduate nursing curriculum in an embedded manner [38]. In-class needs to be combined with the characteristics of the various types of nursing courses to develop professional values in a focused manner. Out-of-class is combined with the implicit curriculum and is not restricted by the materials. It is based on carrying out various types of thematic activities, with the students being the centre and participating actively, giving full play to their subjective initiative. In this study, the education framework is closely integrated with the professional nursing curriculum and the teaching design based on the objectives of the professional curriculum is combined with the professional values development objectives so that the professional values development will have a significant effect on the formation and internalisation of students' professional values.

## Conclusion

This study was a structured and systematic education framework for cultivating professional values among undergraduate nursing students based on preliminary systematic theoretical research. The education framework formed in this study has a solid academic foundation, and the content design takes account of student-centred teaching activities and makes use of available resources to mobilise students' independent participation, and adopts a multi-faceted perspective to comprehensively evaluate the development effects, ultimately forming a professional values education framework for undergraduate nursing students with structural and practical significance, providing educational strategies and methods for internalisation of professional values for nursing students, and enriching the values development strategies in undergraduate nursing education. However, the study has not yet been validated, and our research group will explore the feasibility and practicality of this educational framework through experimental studies in the future.

### Supplementary Information

**Additional file 1:** **Appendix 1. **Search strategy (Pubmed). **Appendix 2.** Demographic characteristics for members of the Expert Meeting. **Appendix 3.** Summary table of included studies (quasi-experimental study and action research). **Appendix 4.** Summary table of included studies (review).

## Data Availability

All data generated or analysed during this study are included in this published article.
