# Supplementary material for: Professional values education for undergraduate nursing students: developing a framework based on the professional values growth theory
Source: BMC Nurs. 2024 Apr 2;23:226. doi: 10.1186/s12912-024-01743-0 (PMC10986104; doi:10.1186/s12912-024-01743-0)
Supplement: Supplementary file 1 — Additional file 1: Appendix 1. Search strategy (Pubmed). Appendix 2. Demographic characteristics for members of the Expert Meeting. Appendix 3. Summary table of included studies (quasi-experimental study and action research). Appendix 4. Summary table of included studies (review). [file 12912_2024_1743_MOESM1_ESM.docx]

**Appendix 1**

Table Search strategy（PubMed）

| **Items** | **Terms** |
| --- | --- |
| P（population） | "students, nursing"[MeSH Terms] |
| C（concept） | "professional values"[Title/Abstract] AND "nurses"[MeSH Terms] |
| C（contest）* | ”qualitative research”[MeSH Terms] OR “factors”[Title/Abstract]OR “review”[Title/Abstract] OR “comparative study”[Title/Abstract] OR “cross-sectional survey”[Title/Abstract] OR ”comparison[Title/Abstract] OR “comparing”[Title/Abstract] OR ” Perspectives”[Title/Abstract]) |
| Search formula | P AND C NOT C |

* This study uses Boolean logic ‘NOT’ to identify context terms.

**Appendix 2**

Table Demographic characteristics for members of the Expert Meeting

| No. | Gender | Age | Work years | Title | Education background | Workplace | Fields |
| --- | --- | --- | --- | --- | --- | --- | --- |
| 1 | female | 69 | 40 | Professor | Ph.D. | College | Nursing education  Health education |
| 2 | male | 60 | 38 | Professor | Ph.D. | College | Medical education  Health and Humanities |
| 3 | female | 61 | 39 | Professor | Ph.D. | College | Nursing Psychology  Nursing Education |
| 4 | female | 56 | 34 | Professor | Ph.D. | College | Evidence-based Nursing |
| 5 | female | 53 | 27 | Professor | Ph.D. | College | Geriatric Nursing |

**Appendix 3**

Table Summary table of included studies（quasi-experimental study and action research）

| **No.** | **Authors（year,**  **country）** | **Type** | **Theoretical foundation** | **Aims** | **Methods** | **Population** | **Instruments** | **Data collection methods** | **Results** |
| --- | --- | --- | --- | --- | --- | --- | --- | --- | --- |
| 1 | Sara White^[149]^（2021，British） | Action Research | Humanistic Values Framework | To understand how nursing students describe their values and their perceptions of the factors that influence these values in a nursing curriculum based on humanistic values. | The study was divided into three phases:in the first phase, VCEs were collected from students (n=161) who participated in the project; in the second phase, the themes developed in the first phase were shared with students and VCEs were collected again (n=82), at which point focus group interviews were used to collect information, with four focus groups of 31 people; in the third phase, collaborative survey groups (n=151, 5 groups) were used to share the results of the second phase with students and answer related questions. | Undergraduate nursing students at a UK university from 2013 to 2016, all of whom participated in a humanistic values-based nursing course | Values Clarification Exercise (VCE) questionnaire | Questionnaire approach; focus group interview method | The three-stage survey reflects the evolution of nursing students' experiences and expectations from professional idealism to the reality of professional practice. It demonstrates how students develop their confidence and resilience by internalising humanistic values. |
| 2 | Heather Ferrillo^[121]^（2020， USA） | quasi-experimental study | Kolb's experiential learning theory | To understand the role of international service learning developing professional values in nursing for nursing students. | The intervention group was to participate in a one-week international service learning programme, an international nursing practice exchange programme in which nurses participate in study tours abroad, conference preparation, clinical simulation, reflective journaling and debriefing. The control group did not participate in the programme. | 76 nursing students at a private university in the USA, 30 in the experimental group and 46 in the control group (originally 48, with 2 drop-outs) | Revised Nursing Professional Values Scale (NPVS-R) | Questionnaire approach | Participants in the intervention group reported higher levels of professional values than those in the control group, but the difference was not statistically significant. |
| 3 | BeverlyWDabney^[152]^（2019、USA） | quasi-experimental study（pretest- posttest design） | American Nurses Association Code of Ethics | To determine nursing students' attitudes and beliefs about professional values when taking online courses (including ethics). | Course Project: RN- to-BSN Online Web Project in the Undergraduate Curriculum: This project is an integration of the American Nurses Association's Code of Ethics and Interpretive Statements for Nurses document by faculty at a public university in the western United States to assign its content to the core baccalaureate curriculum, with a course in ethics taken throughout, which is a total of The three-credit course covers the study of critical frameworks for legal, ethical and moral decision-making in nursing and health care; self-awareness and values clarification by focusing on real-life clinical cases as guidance for registered nurses. | 119 students participated in the program from the spring semester of 2015 - the spring semester of 2018, with students coming from a university | NPVS-R | Questionnaire approach | There was a significant increase in overall post-test scores. Students who took an ethics course after the pre-test showed a considerable increase in their post-test scores, while students who took an ethics course before the pre-test showed a small but statistically insignificant increase in their post-test scores. |
| 4 | Cevriye Yüksel Kaçan^[122]^（2019，Turkey） | Non-randomised controlled trials（pretest posttest design） | / | To explore the impact of transcultural nursing education on nursing students' professional values, empathy skills, cultural sensitivity, and intelligence. | The intervention group receives a transcultural nursing curriculum that focuses on health and culture, nursing and culture, intercultural nursing and historical development, basic concepts of intercultural nursing, intercultural nursing models, ethics in intercultural nursing, and culturally adequate and culturally sensitive approaches in nursing research. The control group did not receive this education. | The intervention group was 65 students enrolled in the Intertextual Nursing course. The control group consisted of students who were not enrolled in this course (n = 60). | NPVS-R | Questionnaire approach | Post-test scores on all scales were significantly higher in the intervention group than in the control group. |
| 5 | Hyangjin PARK^[153]^（2021，Korea） | quasi-experimental study（pretest- posttest design） | A film teaching philosophy that embraces student-centred learning, problem-solving learning, experiential learning, and reflective learning. | (i) To use a film education method to help second-year nursing students develop a correct professional identity in nursing.  (ii) To explore the effects of this method on nursing perceptions, professional satisfaction and nursing professional values. | The nursing education program was developed based on the learning concepts of film education and the core concepts of nursing. The project incorporates requirements through film by including six films that address the following concepts: Me Before You (problem-solving), Testament of Youth (nursing management), Girl, Interrupted (interpersonal skills and nursing knowledge), Hungry Heart (interpersonal skills and problem-solving), Iris (nursing knowledge and problem-solving), and Chronic (Nursing Knowledge and Collaboration). | The experimental group (n=15) participated in an 8-week nursing education programme, and the control group (n=15) did not participate All participants were sophomore nursing students. | Korean version of the Nursing Professional Values Scale | Questionnaire approach | The experimental group showed significantly more improvements in nursing perceptions, professional satisfaction and nursing professional values than the control group, with significant results. |

**Appendix 4**

Table Summary table of included studies（review）

| **No.** | **Authors（year,**  **country）** | **Type** | **Theoretical foundation** | **Aims** | **Contents** |
| --- | --- | --- | --- | --- | --- |
| 1 | Toni M. Vezeau^[156]^（2006、USA） | Review | The five core nursing professional values of the American Association of Colleges of Nursing (AACN) | This study is based on the AACN's five core professional values, which are summarised and elaborated upon, suggesting specific components of values education so that they can be applied more clearly and deeply in nursing education, and identifying challenges for students in the process of forming values. | Step 1: Early introduction of values in nursing education: Values should be introduced early in the nursing curriculum as part of the socialization of students into their professional identity. This can be done in the classroom, laboratory, or clinical setting through lectures, seminars, or clinical sessions. The time to carry out this is the first semester.  Step 2: Motivating students: Understanding their values, expectations of the profession, and the expected reflection of dissonance, students need to understand the nature of health care and its complexity, and value conflicts with clients, families, colleagues, and co-workers. Understand the context of negotiating nursing practice in the context of multiple values.  Step 3: Nature of Values: Understanding the process of values growth and development and the need to learn nursing profession’s values.  Step 4: Cultural Values: Introduce basic cultural concepts and enable students to understand personal values and how professional values illuminate cultural values. Begin to identify those values that are meaningful and relate these values to their own culture.  Step 5: Examine personal values: ask questions to help students identify their values.  Step 6: Identify the AACN's five core professional values |
| 2 | Annette M. Elliott^[157]^（2017. Turkey） | Model Construction | None | To develop a conceptual framework of professional values to improve values-based nursing practice. | The model aims: (i) to describe the characteristics, values, and experiences of individuals to provide care based on the whole person; (ii) to describe the personal and professional values of nurses to help recognize themselves; (iii) to enhance the professional values of nurses to improve their job satisfaction; and to provide high-quality care to improve patient satisfaction with care.  Assumptions: (i) individuals attempt to create a condition in which they can demonstrate their values-related health behaviours; (ii) people are constantly changing and evolving; and (iii) individuals are influenced by their environment at all stages of their lives.  The framework of the model: Three basic concepts: personal values, professional nursing values, and quality of care. Individual traits include physical, psychological, and social characteristics. Within personal values are prior experiences, perceptions of health and illness, needs and priorities; professional values of nursing: truth (adherence to facts or reality), integrity (acceptance of standards of practice by appropriate ethical norms), altruism (welfare of others), autonomy to make choices (ability to decide or act), equality (having the same rights and privileges), human dignity (the intrinsic value of the individual), and aesthetics. intrinsic value); aesthetics (different habits about what is appropriate, tasteful, fashionable, or pleasing, and what we appreciate and value fundamentally); quality of care: job satisfaction and patient satisfaction. |
| 3 | NANCY L^[158]^（2005，USA） | review | AACN Five Core Nursing Professional Values | Describe the content of values-based nursing education | The curriculum strategy includes five semesters of content: (i) semester 1: "Human Dignity" (definition of the concept, prediction of how such values are demonstrated in clinical nursing practice, alternative experiences of sensory deficits in communication courses, etc.); (ii) semester 2: "Integrity" (analysis of disciplinary incidents among nurses, development of professional discretion in community geriatric clinical practice, professional boundaries in clinical practice); (iii) Semester 3: "Autonomy": (promotion of optimal decision-making in clinical practice, students independently helping patients to collect and interpret relevant health information, enabling patients to make informed choices, creating a copy of their Semester 4: "Altruism" (learning about public figures, watching films and finding stories of altruistic behaviour by themselves and others); 5. international social justice issues related to health, sharing and discussing issues related to social justice experienced in clinical placement exchange sessions, public health nursing courses, discussing public health issues, and watching relevant films.) |
| 4 | Sandra Lynch^[159]^（2013，Australia） | review | The seven pillars and twelve assumptions in the GVV and the Macula model of ethical reasoning developed earlier. | The course is designed to help students and professionals explore and practise responses when faced with conflicting values and beliefs, ensuring they can respond to their importance in complex work situations. | The GVV (Happening for Values) programme consists of seven 'pillars' and twelve assumptions: values, choices, norms, purpose, self-awareness and alignment, voice, reason, and rationalisation. Within the context of care, the seven pillars of choice, standards, self-awareness, and alignment have attracted the attention of educators. Nursing students were progressively trained in self-awareness of their values through group discussions, story sharing, exercises, and coaching during the practice. At the same time, the project was evaluated at the end of its duration and the results were divided into three themes: knowledge of values, the discourse of values, and action of values. |
| 5 | Christopher McLea^[160]^（2011，British） | review | / | The development of a values-based model of inquiry learning and teaching | An ideal introduction and literature review summarise the development of a values-based inquiry learning and teaching model. The model includes the core three concepts of self-awareness, professional values of care and compassion, awareness of the values of others, and three sets of prompting questions developed around model's core to guide learning and development further.. |
